# State medical malpractice laws and utilization of surgical treatment for rotator cuff tear and proximal humerus fracture: an observational cohort study

**DOI:** 10.1186/s12913-021-06544-8

**Published:** 2021-05-28

**Authors:** Brian Chen, Cole Chapman, Sarah Bauer Floyd, John Mobley, John Brooks

**Affiliations:** 1 Department of Health Services Policy and Management, University of South Carolina, 915 Greene Street Suite 354, Columbia, SC 29205 USA; 2 Department of Pharmacy Practice and Science, University of Iowa, 345 CPB, 180 South Grand Ave, Iowa City, IA 52242 USA; 3 College of Behavioral, Social and Health Sciences, Clemson University, 116 Edwards Hall, Clemson, SC 29634 USA; 4 University of South Carolina School of Medicine Greenville , 607 Grove Rd, SC 29605 Greenville, USA; 5 Department of Health Services Policy and Management, University of South Carolina, 915 Greene Street Suite 302, Columbia, SC 29205 USA

**Keywords:** Medical malpractice, Defensive medicine, Orthopaedics, Medicare

## Abstract

**Background:**

How much does the medical malpractice system affect treatment decisions in orthopaedics? To further this inquiry, we sought to assess whether malpractice liability is associated with differences in surgery rates among elderly orthopaedic patients.

**Methods:**

Medicare data were obtained for patients with a rotator cuff tear or proximal humerus fracture in 2011. Multivariate regressions were used to assess whether the probability of surgery is associated with various state-level rules that increase or decrease malpractice liability risks.

**Results:**

Study results indicate that lower liability is associated with higher surgery rates. States with joint and several liability, caps on punitive damages, and punitive evidence rule had surgery rates that were respectively 5%-, 1%-, and 1%-point higher for rotator cuff tears, and 2%-, 2%- and 1%-point higher for proximal humerus fractures. Conversely, greater liability is associated with lower surgery rates, respectively 6%- and 9%-points lower for rotator cuff patients in states with comparative negligence and pure comparative negligence.

**Conclusions:**

Medical malpractice liability is associated with orthopaedic treatment choices. Future research should investigate whether treatment differences result in health outcome changes to assess the costs and benefits of the medical liability system.

**Supplementary Information:**

The online version contains supplementary material available at 10.1186/s12913-021-06544-8.

## Introduction

Does the medical malpractice system affect the practice of medicine? We ask this question in the context of orthopaedics, a medical specialty that accounts for $176 billion in direct annual healthcare expenditures and affects one in every two Americans over age 18 [1–3]. Orthopaedics is also a field with substantial physician discretion because very little level I evidence exists to guide treatment choices [4–19]. Orthopaedics is also a high-risk specialty, and orthopaedic surgeons are among the most commonly sued physicians for medical malpractice, with a high ratio of claims paid out to claims made [20]. As a result, orthopaedic surgeons may be especially sensitive to medical malpractice liability regimes when making their treatment recommendations, with far-reaching implications for healthcare costs and patient outcomes.

Debate over the desirability of medical malpractice laws remains a contentious issue. In addition to the direct costs of litigation, medical malpractice may increase costs indirectly through “defensive medicine,” defined as medical practices that reduce legal liability without significant benefit to patients [21, 22]. For example, physicians may order diagnostic tests such as CAT scans, MRIs and X-rays when such actions are not likely to improve patient health (“positive defensive medicine”) [23]. The fear of liability may also discourage physicians from performing medically necessary but high-risk procedures (“negative defensive medicine”) [24]. Concerns over the adverse cost impact of defensive medicine ultimately provided the political impetus for medical malpractice liability reforms (and more generally, tort reforms) at the state level in the late 1990s.^1^ These reforms primarily limited awards to patients or created procedural barriers for patients to sue their physicians [29].

The objective of this study was to empirically assess whether state tort regimes are associated with surgery rates for two common shoulder conditions to explore defensive medicine in orthopaedics. Our hypothesis is that tort rules that lower legal liability could encourage physicians to undertake greater risk and therefore increase utilization of orthopaedic surgeries. We also hypothesize the converse, that tort rules that increase liability are associated with reductions in orthopaedic surgical procedures.

Our study contributes to the vast literature on medical malpractice in several ways. Existing research has shown little consistent relationship between tort liability and measures of defensive medical practices [30, 31], possibly because studies investigate different medical contexts. Most studies focus on obstetrics/gynecology [32–49] or chronic conditions such as heart disease [50–52], neurology [53–58], or Medicare patients in general [59–61]. Our study instead focuses on orthopaedics, an important medical specialty that is understudied in the medical malpractice literature, and is among the medical specialties with the highest risk for medical malpractice claims.

Methodologically, few studies in orthopaedics go beyond survey methods to assess the impact of tort liability on physician treatment choices [21, 62–65]. Beyond orthopaedics, many studies also simply surveyed physicians [21, 38, 39, 53, 55, 62, 66–68], an approach that is likely to elicit self-interested reporting bias from physicians. We will adopt a more objective measure by examining treatment choices in claim databases.

Finally, most studies evaluating the effects of tort liability focus on establishing positive defensive medicine [21, 52, 60–62, 66, 67, 69–71]. By assessing avoidance behavior, our work is also important because of the social burden resulting from avoiding patients requiring care in response to different liability regimes. Our results are the first step to help calibrate the need to balance the tradeoff between patient compensation for iatrogenic injury and the law’s undue influence on physician decision-making.

## Methods

### Data and sample

Data for our study included complete Medicare claims and beneficiary summary files from years 2010–2012 for all Medicare patients in the United States with any physician service for one of 192 shoulder-related diagnoses in 2011. To identify incident diagnoses, beneficiaries with any shoulder-related diagnosis within 365-days prior to their index shoulder diagnosis in 2011 were excluded. Beneficiaries diagnosed with proximal humerus fracture (PHF) or atraumatic rotator cuff tear (ARCT) with an index date in 2011 were then identified using specific algorithms described below. Data from 2010 were used to measure baseline patient characteristics and to exclude patients with non-incident shoulder conditions in 2011, and data from 2012 were assessed to identify subsequent use of surgery. This project was approved by the University of South Carolina Institutional Review Board.

### Proximal Humerus fracture sample

The PHF sample was identified by International Classification of Diseases, Ninth Revision, Clinical Modification (ICD-9-CM) diagnosis codes: 812.00, 812.01, 812.02, 812.09, 812.10, 812.11, 812.12, 812.13, and 812.19. The first observed diagnosis date in 2011 was defined as the index date. Beneficiaries with no X-ray of the shoulder or upper extremity within 7-days of their index PHF diagnosis were excluded to limit the potential for misdiagnosis.

### Atraumatic rotator cuff tear sample

Presence of ARCT cannot be confirmed without radiologic evidence or surgery. To limit misdiagnosis, patients with no MRI within 90-days of the date of initial shoulder problem in 2011 were excluded. Then, patients with no ICD-9-CM diagnosis code indicating ARCT within 14 days after the date of MRI were excluded. Patients were coded to have received surgery if surgery occurred before any physical therapy in claims during the 104-days (90 + 14) after the index diagnosis date.

Additional inclusion criteria applied to both clinical samples included (1) continuous enrollment in fee-for-service Medicare Part A and Part B, and no enrollment in Medicare Part C, during the study period from 365 days prior to 365 days after the index diagnosis or death; (2) aged 66 years or older on their initial shoulder diagnosis date in 2011; (3) residence within the United States; and (4) complete geographic location information. Full inclusion criteria for both clinical samples are provided in Additional file 1: Appendix A1.

### Dependent variables

#### Treatment

Treatment windows were defined in the 60-day period following the index PHF diagnosis and 104-day period following the ARCT index event. Patients receiving one of the surgical treatments for fracture (hemiarthroplasty, reverse arthroplasty, open reduction internal fixation) or ARCT (open or arthroscopic rotator cuff repair) were classified as surgery patients. Patients receiving no surgery in the treatment observation window were classified as conservative management patients. Surgery claims were identified using ICD-9-CM Procedure codes and Healthcare Common Procedure Coding System (HCPCS) codes in Medicare Outpatient, MEDPAR, and Carrier files. Complete definitions for all variables are provided in the Additional file 1: Appendix A2.

### Independent variables

#### State-level tort Laws

We obtained data on state tort law liability regimes from the Database of State Tort Law Reforms, 1980–2018 (6th Edition) from the University of Texas School of Law [72]. The key explanatory variables of interest are state-level measures of malpractice and tort rules, in three general categories: 1) attribution of fault, 2) limits on liability, and 3) rules of evidence. All of the reform rules (except the comparative negligence rules) decreased physician malpractice liability pressure. Because most of the tort rules that we study were implemented beginning in the late-1990s, we estimated the long-term, stable effects of differences in tort liability rules across states.

**Attribution of fault** determines how fault is apportioned between multiple defendants (joint and several liability rule) or between defendant and plaintiff when both parties are partially at fault (contributory/comparative negligence). *Joint and several liability rule* (JSL) is coded as 1 if a state requires patients to sue providers most at fault for their harm rather than the medical team as a whole. For any individual provider, JSL reduced liability pressure because modern medicine is so complex that patients may not necessarily be able identify the correct party to sue.

When both providers and patients are at fault, “*comparative negligence*” is coded as 1 if a state requires that physicians be at least as much at fault (> = 50% at fault) as the patients in order to be liable. “*Pure comparative*” set to 1 means that a plaintiff can recover from a defendant, even if the plaintiff is much more at fault than the defendant. Unlike other tort rules in our study, the comparative negligence and pure comparative negligence rules *increase* physician liability pressure by making it possible for patients to recover damages even when patients are partially fault. Prior to the reforms, patients could not recover from a negligent doctor if they were even slightly at fault (“contributory negligence”).

**Physician liability** rules dictate how much monetary liability that a physician at fault faces. “*Cap on non-economic damages*” coded as 1 means that there is a cap on monetary damages for non-physical harm, primarily “pain and suffering.” “*Cap on punitive damages*” places a limit on how much patients can receive for physicians’ unconscionable behavior that goes beyond mere negligence.

**Rules of evidence** instruct the jury to consider additional factors when weighing the evidence for monetary awards. “*Punitive evidence rule*” is coded as 1 if the evidence against extremely negligent physicians must be substantial for them to be held liable. “*Collateral source rule*” coded as 1 means that juries could obtain information on whether patients were compensated elsewhere (e.g., workers’ compensation) to consider when determining the award amount.

The status of the tort rules relevant for each patient was determined based upon each patient’s state of residence reported to Medicare in 2011. See Table 1.

**Table 1:** Status of Tort Liability Rules by State, 2011

	Attribution of fault				Physician liability		Evidence	
	JSL Reform	Contributory negligence	Comparative negligence	Pure comparative negligence	Cap on non-economic damages	Cap on punitive damages	Punitive evidence	Collateral source rule
Alabama	No	Yes	No	No	No	Yes	Yes	Yes
Arizona	Yes	No	No	Yes	No	No	Yes	Yes
Arkansas	Yes	No	Yes	No	No	Yes	Yes	No
California	Yes	No	No	Yes	Yes	No	Yes	Yes
Colorado	Yes	No	Yes	No	Yes	Yes	Yes	Yes
Connecticut	Yes	No	Yes	No	No	No	No	Yes
Delaware	No	No	Yes	No	No	No	Yes	Yes
Dist. of Columbia	No	Yes	No	No	No	No	Yes	No
Florida	Yes	No	No	Yes	Yes	Yes	Yes	Yes
Georgia	Yes	No	Yes	No	No	Yes	Yes	No
Idaho	Yes	No	Yes	No	Yes	Yes	Yes	Yes
Illinois	No	No	No	Yes	No	Yes	No	Yes
Indiana	No	No	Yes	No	No	Yes	Yes	Yes
Iowa	Yes	No	Yes	No	No	No	Yes	Yes
Kansas	Yes	No	Yes	No	Yes	Yes	Yes	No
Kentucky	Yes	No	No	Yes	No	No	No	No
Louisiana	Yes	No	No	Yes	No	Yes	No	No
Maine	No	No	Yes	No	No	No	Yes	Yes
Maryland	No	Yes	No	No	Yes	No	Yes	No
Massachusetts	No	No	Yes	No	Yes	No	No	Yes
Michigan	Yes	No	No	Yes	Yes	Yes	No	Yes
Minnesota	Yes	No	Yes	No	No	No	Yes	Yes
Mississippi	Yes	No	No	Yes	Yes	Yes	Yes	No
Missouri	Yes	No	No	Yes	Yes	Yes	Yes	No
Montana	Yes	No	Yes	No	Yes	Yes	Yes	Yes
Nebraska	Yes	No	Yes	No	No	Yes	No	Yes
Nevada	Yes	No	Yes	No	Yes	Yes	Yes	Yes
New Hampshire	Yes	No	Yes	No	No	Yes	No	No
New Jersey	Yes	No	Yes	No	No	Yes	Yes	Yes
New Mexico	Yes	No	No	Yes	No	No	No	No
New York	Yes	No	No	Yes	No	No	No	Yes
North Carolina	No	Yes	No	No	No	Yes	Yes	No
North Dakota	Yes	No	Yes	No	Yes	Yes	Yes	Yes
Ohio	Yes	No	Yes	No	Yes	Yes	Yes	Yes
Oklahoma	Yes	No	Yes	No	Yes	Yes	Yes	Yes
Oregon	Yes	No	Yes	No	No	Yes	Yes	Yes
Pennsylvania	N/A	No	Yes	No	No	Yes	Yes	Yes
Rhode Island	No	No	No	Yes	No	No	No	Yes
South Carolina	Yes	No	Yes	No	Yes	No	Yes	No
South Dakota	Yes	No	Yes	No	Yes	No	Yes	Yes
Tennessee	Yes	No	Yes	No	No	No	Yes	Yes
Texas	Yes	No	Yes	No	Yes	Yes	Yes	No
Utah	Yes	No	Yes	No	Yes	No	Yes	Yes
Vermont	Yes	No	Yes	No	No	No	No	No
Virginia	No	Yes	No	No	No	Yes	No	No
Washington	Yes	No	No	Yes	No	Yes	No	Yes
West Virginia	Yes	No	Yes	No	Yes	No	No	Yes
Wisconsin	Yes	No	Yes	No	Yes	Yes	Yes	Yes
Wyoming	Yes	No	Yes	No	No	No	No	No

#### Patient clinical and demographic characteristics

Patient demographic characteristics were identified using Medicare’s 2011 Beneficiary Summary. Specific patient-level variables included age at index, sex, race, and state of residence reported to Medicare in 2011, and Medicaid dual-eligibility status during the index month. Patient health status and comorbidity were measured using the Charlson Comorbidity Index [73–75] and Frailty Risk Index [76]. Both indices were measured using data from 365 days prior to index. Total Medicare reimbursements by patient over the 365-day period prior to index were placed into quartiles to capture further differences in comorbidity and health status, as well as differences in healthcare-seeking behaviors.

### Analytical approach

This study assessed whether differences in state-level tort rules in 2011 were statistically associated with receipt of surgery as treatment. Our primary approach was to regress the binary variable indicating surgery receipt on patient-level demographic and clinical characteristics and state-level indicators of malpractice and tort laws. We ran separate models for PHF and ARCT patients. The contribution of each state-level malpractice and tort law was assessed by examining their respective parameter estimates and 95% confidence intervals, as well as by the F-statistic from comparisons with a model excluding the relevant variable. In addition, we reported robust standard errors clustered at the state level.

We chose the linear probability model to estimate and report the associations between tort rules and binomial outcomes because with our large sample size and the Central Limit Theorem, this method enables us to test our regression coefficient using the fewest assumptions related to the error term. According to the Central Limit Theorem, linear regression coefficients are distributed normally when sample size (n) times the probability of the outcome occurring (p) > 5 and n(1-p) > 5 [77]. Both conditions are easily satisfied with the smallest size of our analyses stratum *n* = 32,163 and the surgery rate of p = approximately 20%. In addition, the coefficients that we report from the linear probability model can be interpreted directly as the percentage-point difference in probability of surgery associated with each tort liability rule. Further, our linear probability models were estimated using robust standard errors to alleviate heteroscedasticity concerns [78].

We used SAS software version 9.4 (Cary, NC, USA) to create the analytical samples from the full Medicare dataset, and R version 3.5.2 (Vienna, Austria) to conduct the statistical analyses.

## Results

In Table 2, we present the summary statistics of key variables in our analytic samples. In our ARCT sample, we had a total of 32,163 individual patients based on our inclusion and exclusion criteria. The corresponding figure is 77,053 for PHF patients. Ultimately, 19.85 and 15.36% of ARCT and PHF patients respectively received surgery within the follow-up periods. While patients with ARCT were almost evenly split between men (50.23%) and women (49.77%), PHF patients were overwhelmingly female (80.11%). The remaining summary statistics demonstrate that PHF patients were more likely to have more complex conditions or more vulnerable than ARCT patients, suggesting that physicians likely have greater discretion with treatment choices for ARCT patients. In our data, Medicare expenditures in the year prior to index diagnosis averaged $15,622.73 for PHF patients but only $7593.52 for ARCT patients. Twice as many PHF versus ARCT patients were dually eligible for Medicare/Medicaid (17.33% for PHF versus 6.84% for ARCT). PHF patients were on average approximately 6 years older (80.26 versus 73.97 years old), and were approximately four times more likely to have the highest score of frailty (24.11% for PHF versus 5.80% for ARCT). In addition, the average Charlson Comorbidity Index at baseline was 2.57 for PHF patients, and 1.59 for ARCT patients.

**Table 2:** Summary statistics of analytical samples

	Atraumatic Rotator Cuff Tear				Proximal Humerus Fracture			
Variable	%	Std Dev	Min	Max	%	Std. Dev.	Min	Max
	N = 32,163				*N* = 76,648			
Surgery within follow-up	19.85%				15.36%			
Male	50.23%				19.89%			
Dually eligible (full or partial)	6.84%				17.33%			
Black	4.27%				3.13%			
Hispanic	1.25%				1.35%			
Other	2.39%				2.14%			
White	92.10%				93.37%			
Age	73.97	5.80	66.00	100.31	80.26	8.07	66.00	115.46
Baseline expenditures	7593.52	14,086.28	0.00	621,235.40	15,622.73	26,557.62	0.00	878,736.70
Baseline frailty 0	61.16%				34.14%			
Baseline frailty 1	24.95%				25.90%			
Baseline frailty 2	8.09%				15.85%			
Baseline frailty 3	5.80%				24.11%			
Baseline Charlson Index	1.59	1.97	0.00	18.00	2.57	2.70	0.00	24.00

### Tort-related results

Our results corroborate the hypothesis that lower malpractice liability is associated with greater utilization of surgical procedures for ARCT and PHF. See Table 3 for ARCT (Panel A). States that had JSL rules (lower liability) had a higher surgery rates by 5% points (CI 0.03–0.06, *p* < 0.01). Similarly, states with a cap on punitive damages or higher levels of evidence required for punitive damages also had higher surgery utilization rates, respectively by 1% point (CI 0.00–0.02, *p* < 0.05) and 1% point (CI 0.00–0.03, *p* < 0.05).

**Table 3:** Association between legal rules and surgery among Medicare patients with ARCT or PHF

	Panel A: ARCT			Panel B: PHF		
*Predictors*	*Estimates*	*95% CI*	*p*	*Estimates*	*95% CI*	*p*
(Intercept)	0.21	0.16–0.26	**< 0.001**	0.19	0.16–0.22	**< 0.001**
***Tort Law Variables***						
JSL rule	0.05	0.03–0.06	**< 0.001**	0.02	0.01–0.03	**< 0.001**
mod comparative	-0.06	-0.08 – −0.04	**< 0.001**	-0.01	-0.02 – −0.00	**0.031**
pure comparative	−0.09	−0.11 – −0.06	**< 0.001**	0	−0.01 – 0.01	0.809
cap nonec	−0.01	−0.02 – 0.00	0.146	0	−0.00 – 0.01	0.541
cap puni	0.01	0.00–0.02	**0.011**	0.02	0.02–0.03	**< 0.001**
puni evid	0.01	0.00–0.03	**0.016**	0.01	0.01–0.02	**< 0.001**
coll source	− 0.01	− 0.02 – 0.01	0.316	0	− 0.01 – 0.00	0.323
***Control Variables***						
male	0.04	0.03–0.05	**< 0.001**	− 0.02	− 0.03 – − 0.02	**< 0.001**
dual elig any	− 0.04	− 0.06 – − 0.02	**< 0.001**	− 0.04	− 0.05 – − 0.03	**< 0.001**
race Black	0.03	− 0.02 – 0.08	0.217	− 0.04	− 0.07 – − 0.00	**0.025**
race Hispanic	0.02	− 0.04 – 0.08	0.549	0	− 0.03 – 0.04	0.833
race Other	0.05	− 0.01 – 0.11	0.080	− 0.01	− 0.04 – 0.03	0.687
race White	0.07	0.02–0.12	**0.004**	0.02	− 0.01 – 0.04	0.27
age group (70–75)	−0.02	− 0.03 – − 0.01	**< 0.001**	-0.01	− 0.02 – 0.00	0.082
age group (76–79)	− 0.07	− 0.08 – − 0.05	**< 0.001**	−0.01	− 0.02 – − 0.00	**0.003**
age group (80–85)	−0.12	− 0.13 – − 0.10	**< 0.001**	−0.05	− 0.06 – − 0.04	**< 0.001**
age group (86+)	−0.14	− 0.17 – − 0.12	**< 0.001**	−0.10	− 0.11 – − 0.09	**< 0.001**
cost pre 365 (Q2)*	−0.02	− 0.03 – − 0.00	**0.013**	0	-0.00 – 0.01	0.425
cost pre 365 (Q3)*	−0.03	−0.05 – − 0.02	**< 0.001**	0.01	− 0.00 – 0.02	0.076
cost pre 365 (Q4)*	−0.04	− 0.05 – − 0.03	**< 0.001**	0	− 0.01 – 0.01	0.994
FRI (1)	− 0.01	− 0.02 – 0.00	0.093	− 0.02	− 0.03 – − 0.01	**< 0.001**
FRI (2)	− 0.02	−0.03 – 0.00	0.064	−0.03	− 0.03 – − 0.02	**< 0.001**
FRI (3+)	−0.04	− 0.06 – − 0.01	**0.001**	−0.05	− 0.06 – − 0.04	**< 0.001**
cci group (1)	− 0.01	−0.02 – 0.00	0.104	−0.01	− 0.02 – 0.00	0.07
cci group (2)	−0.02	−0.03 – − 0.00	**0.023**	−0.02	− 0.02 – − 0.01	**< 0.001**
cci group (3)	− 0.02	−0.04 – − 0.01	**0.007**	-0.01	− 0.02 – − 0.00	**0.019**
cci group (4+)	− 0.04	−0.05 – − 0.02	**< 0.001**	-0.02	− 0.03 – − 0.02	**< 0.001**
Observations	32,163			76,648		
R2 / R2 adjusted	0.032 / 0.031			0.023 / 0.023		

*Q2, Q3, Q4 refer to quartiles

Conversely, higher provider liability risk is associated with lower surgery rates following diagnosis of ARCT. Comparative negligence and pure comparative negligence both increase physician liability risks relative to those in states that adhere to contributory negligence. Patients in comparative negligence states have 6%-point lower probability (CI -0.08 – − 0.04, *p* < 0.01) and those in pure comparative negligence states, 9%-point lower probability (CI -0.11 – − 0.06, *p* < 0.01) of undergoing surgery.

We present our results for PHF in Table 3 (Panel B). Again, JSL rule is associated with an increase in surgeries among PHF patients by 2% points (CI 0.01–0.03, *p* < 0.01). States with caps on punitive damages and higher evidentiary requirement for punitive damages are associated with a 2%-point (CI 0.02–0.03, *p* < 0.01) and 1%-point (CI 0.01–0.02, *p* < 0.01) increase in the probability of surgery. For PHF, the comparative negligence rules have a weaker statistical relationship with receipt of surgery, but in the hypothesized direction (a decrease of 1%-point (− 0.02 – − 0.00, *p* < 0.05) in the probability of surgery).

### Results related to clinical and patient demographic variables

While we included patient-level demographic and clinical characteristics primarily as control variables, the results are worth exploring as well. Aside from sex, in both patient samples, all other statistically significant coefficients suggest that patients with more complex conditions or in other ways more vulnerable are less likely to receive surgery. Medicare-Medicaid dual eligible patients are less likely to receive surgery by 4%-points (CI -0.06 – − 0.02, *p* < 0.01) for ARCT and by 4%-points (CI -0.05 – − 0.03, *p* < 0.01) for PHF. Older patients are respectively less likely to undergo surgery for ARCT by 2% points (CI -0.03 – − 0.01, *p* < 0.01, patients 70–75), 7% points (CI − 0.08 – − 0.05, *p* < 0.01, patients 76–79), 12% points (CI -0.13 – − 0.10, *p* < 0.01, patients 80–85), and 14% points (CI -0.17 – − 0.12, *p* < 0.01, patients 85 and above). The corresponding figures are 1% point (− 0.02–0.00, not statistically significant), 1% point (CI -0.02 – − 0.00, *p* < 0.01), 5% points (CI -0.06 – − 0.04, *p* < 0.01), and 10% points (CI -0.11 – − 0.09, *p* < 0.01) for PHF patients.

Other measures of patient severity corroborate these results. It is reasonable to assume that greater healthcare expenditures and higher Comorbidity Index or Frailty scores at baseline indicate greater patient severity. For ARCT, the top (most severe) quartile or index score is associated with a reduction in surgery, respectively by 4% points (CI -0.05 – − 0.03, *p* < 0.01, highest baseline expenditures), 4% points (CI -0.06 – − 0.01, *p* < 0.01, highest frailty index), and 4% points (CI -0.05 – − 0.02, *p* < 0.01, highest Charlson Comorbidity Index). Likewise for PHF, higher levels of frailty and Charlson Index are both associated with lower probabilities of surgery: there was a 5%-point (CI -0.06 – − 0.04, *p* < 0.01) reduction for frailty group 3, and a reduction of 2%-points (CI -0.03 – − 0.02, *p* < 0.01) for Charlson Index 4.

## Discussion

Our findings are consistent with the hypothesis that provider tort liability is associated with use of higher-risk procedures (surgeries). Specifically, higher malpractice pressure (in states with comparative negligence rules) is associated with a lower probability of surgery following an index diagnosis of ARCT and PHF, although the evidence is weaker for the latter. Conversely, lower malpractice pressure (in states with JSL, cap on damages and stricter punitive damage evidentiary rule) is associated with higher probability of surgery for both ARCT and PHF. Overall, our results show that tort liability rules are associated with between a 1%- to 9%-point difference in surgery rates for these conditions.

Our results extend existing studies by showing that negative, not just positive defensive medicine may exist in a previously understudied but important medical specialty. Existent literature primarily shows a positive association between higher liability pressure and greater use of diagnostic tests [21, 46, 62, 67, 79, 80]. Specific to orthopaedics, Sethi et al. (2010) found that imaging tests ordered primarily due to liability concerns may exceed $2 billion per year [21]. Our study adds to the literature by showing that orthopaedic surgeons may also increase surgery when the malpractice pressure is lower. This is significant because of the increased possibility of adverse events when a determining factor for riskier surgeries is the malpractice pressure that surgeons face.

Yet, not all studies find evidence of defensive medicine. The inconsistencies in the literature may also be due to different clinical contexts. It is theoretically possible that defensive practices may be greater when substantial discretion in physician treatment choices exists. While we did not test this hypothesis due to a lack of a measure of discretion, it is suggestive that a greater response to joint and several liability rule occurred with ARCT than with PHF (5% versus 2%), with the former (a muscle tear) likely providing more discretion in treatment choices than the latter (a bone fracture). There was also a larger response to attribution of fault rules when considering the generally younger and less vulnerable ARCT patients (− 6% for comparative negligence and − 9% for pure comparative negligence) rather than in PHF (− 1% for modified comparative negligence).

Differences in the measures of malpractice pressure may also account for the range of findings in the literature. Studies that use measures that are more direct than tort reform rules, such as past litigation experience, malpractice premium levels, or elicit perception of malpractice fears directly at the individual physician level, may in fact observe larger associations between tort liability pressure and defensive practices. A recent study, for example, estimated that 20% of spending for imaging may be due to malpractice concerns by matching physician responses on their perceived malpractice risks and their actual spending or by using physician’s recent litigation experience to proxy for malpractice pressure [59, 66]. Our median results estimating the effect of malpractice fears on surgery utilization are much smaller, ranging from 2 to 4%, possibly due in part to our state-level rather than individual-level measurement of tort liability pressure.

We now turn to non-tort law factors that affect treatment choice. For both ARCT and PHF, measures of socioeconomic vulnerability and illness severity not only have larger point estimates than the tort variables, they are also collectively larger than the tort variables as a whole. Dual eligibility, older age, greater frailty and Charlson Comorbidity Index together account for a 56%-point of differences in the probability of surgery, versus 22% for all tort variables in the context of ARCT (when considering only statistically significant relationships). The corresponding numbers are 31% and 6% for PHF. This finding suggests that physician decision-making is primarily based on patient-specific factors and may only secondarily or minimally be affected by the tort environment.

Overall, viewed in the context of the defensive medicine literature, our results have several implications for policy makers. First, defensive medicine likely exists, but it would be inaccurate to view tort law as purely detrimental. Balancing the intended (reduction in negligence and better health outcomes) and unintended consequences (defensive medicine) is difficult, but categorically restricting patients’ ability to seek compensation from negligent doctors may exacerbate the problem of under-compensation among patients who suffered from medical malpractice.

Second, it is important to recognize that the tort liability regime may play only a comparatively small role in driving medical expenditures. Many of the high estimates cited in the literature and popular press are based on extrapolation or physician surveys. The Congressional Budget Office, Mello and colleagues [81], and Rothberg and colleagues [82] all estimate a much lower contribution of “defensive medicine” to healthcare spending, at 0.02, 1.8 and 2.9% respectively.^2^ Our study, which shows an *increase* in surgeries when liability pressure is reduced, further shows that tort rules that limit liability may in some areas counterintuitively increase expenditures.

### Limitations

There are several limitations to our work that should be noted. First, because of our cross-sectional design, reverse causality is a concern – that is, states that chose to reform may have had lower surgical rates to begin with because of high malpractice pressure. However, the reform rules whose effects we estimated occurred over 20 years prior, so physicians’ surgical rates likely had sufficient time to adjust to the legal rules that were adopted so long ago. On the other hand, the push for tort reform peaked in the late 1990s to early 2000s, so the impact of the legal rule changes may have attenuated since their implementation. We did nevertheless find an effect. Third, a complete analysis of defensive medicine requires studying the effects of tort rules both on utilization and on health outcomes. In this study, we assessed only the former, but we believe that it is important in itself to understand whether tort rules affect behavior. Fourth, the decision to undergo surgery is affected by a host of factors, including both surgeon and patient preferences, both in turn shaped by the external policy environment. In this article, we focused on one aspect of the policy environment that may affect treatment choice – the tort liability regime. However, other policy factors may also influence the decision to perform surgery, including informed consent rules that require greater shared decision-making. These rules may well differ across states, but violating informed consent is a medical malpractice issue for which all of our tort liability rules will determine the financial impact on the surgeon. As a general rule, states with multiple tort reform rules tend to have lower overall restrictions on physician practice choices. Taken together, our study results can therefore also be interpreted as the marginal difference in surgery rates between states that have a less strict versus stricter regulatory environment overall. Finally, regression analyses sometimes produce statistically significant results by chance. However, it is not likely that the same regression specification produces multiple statistically significant results, all in the hypothesized directions, simply by chance.

## Conclusion

Our study suggests that physicians respond to tort liability rules in orthopaedics. Controlling for patient sociodemographic and clinical factors, patients in states with tort rules that lower medical liability risks are more likely to have surgery following an incident diagnosis of ARCT and PHF. Conversely, states with tort rules that increase liability risks are less likely to have surgery for ARCT and PHF. However, our work shows that patient-level socioeconomic and clinical factors collectively affect the choice to undergo surgery more than the tort-related variables. Policymakers and state legislators should consider the real cost of defensive medicine when weighing the tradeoff between the desired and unintended consequences of a tort liability system designed to prevent negligence and compensate victims for physician-induced harm.

## Supplementary Information

**Additional file 1: Appendix 1.** Inclusion and Exclusion Criteria. **Appendix 2.** Full Description of Variables.

## Data Availability

The Medicare claims data underlying our study can be obtained from ResDAC upon successful application, at https://www.resdac.org. The Database of State Tort Law Reforms, 1980–2018 (6th Edition) from the University of Texas School of Law can be accessed at https://law.utexas.edu/faculty/ravraham/dstlr.php.

## Abbreviations

ARCT: Atraumatic Rotator Cuff Tear
CAT: Computerized Axial Tomography
CI: Confidence Intervals
HCPCS: Healthcare Common Procedure Coding System
ICD-9-CM: International Classification of Diseases, Ninth Revision, Clinical Modification
JSL: Joint and Several Liability
MRI: Magnetic Resonance Imaging
MEDPAR: Medicare Provider Analysis and Review
PHF: Proximal Humerus Fracture

## Footnotes

1 Research has shown that the cost of “defensive medicine” is fairly limited. However, in the period leading to the wave of state tort reforms, the common perception was that frivolous medical malpractice lawsuits against physicians increased healthcare expenditures and clogged the courts. Legal reforms to limit medical malpractice claims were therefore thought to provide a solution to the perceived impending crisis. Subsequent studies, however, suggest that the reality is far more nuanced. In a comprehensive review, Sloan and Chepke [25] argued that four discrete segments (legal, medical malpractice insurance, medical care, and government activity) interact to result in a highly complex medical malpractice environment. Avraham et al. [26] found, for example, that some tort reforms reduced insurance premiums, but only for employer-sponsored plans rather than capitated HMO plans [26]. Friedson [27] concluded that caps on noneconomic damages reduced insurance reimbursements for certain procedures, but providers did not reduce charges to insurers . Thorpe [28] reported that state with caps on malpractice claims had insurance premiums that were 17.1% lower than states without caps. Still, Thorpe questioned whether these reductions were consistent with other goals promoted by the medical liability system. Thus, while perceptions may have provided the political impetus to drive tort liability reforms, the results of the reforms are far from uniform and highly context-dependent.

2 For the CBO and Mello figures, the estimated annual costs of defensive medicine were divided by total national healthcare expenditures in 2008, the year in which the studies were conducted. Rothberg et al. reported the percentage figure directly in their manuscript.
