# Supplementary material for: State medical malpractice laws and utilization of surgical treatment for rotator cuff tear and proximal humerus fracture: an observational cohort study
Source: BMC Health Serv Res. 2021 May 28;21:516. doi: 10.1186/s12913-021-06544-8 (PMC8161917; doi:10.1186/s12913-021-06544-8)
Supplement: Supplementary file 1 — Additional file 1: Appendix 1. Inclusion and Exclusion Criteria. Appendix 2. Full Description of Variables. [file 12913_2021_6544_MOESM1_ESM.docx]

**State medical malpractice laws and utilization of surgical treatment for rotator cuff tear and proximal humerus fracture: An observational cohort study**

**Brian Chen*^,^****

915 Greene Street Suite 354, Columbia, SC 29205 USA

Department of Health Services Policy and Management, University of South Carolina

**Cole Chapman***

345 CPB, 180 South Grand Ave, Iowa City, Iowa 52242 USA

Department of Pharmacy Practice and Science, University of Iowa

**Sarah Bauer Floyd**

116 Edwards Hall, Clemson, SC 29634

College of Behavioral, Social and Health Sciences, Clemson University

**John Mobley**

607 Grove Rd, Greenville, SC 29605, SC 29205 USA

University of South Carolina School of Medicine Greenville

**John Brooks**

915 Greene Street Suite 302, Columbia, SC 29205 USA

Department of Health Services Policy and Management, University of South Carolina

*Joint First Authors

**Corresponding Author. Please send all correspondences to [bchen@mailbox.sc.edu](mailto:bchen@mailbox.sc.edu)

**Appendices**

**Appendix A1: Inclusion and Exclusion Criteria**

*Medicare 2011 PHF Sample Inclusion Criteria*

| **Inclusion Criteria** | **N** |
| --- | --- |
| Medicare Part B carrier (physician services), outpatient, or MEDPAR (inpatient) claims with a proximal humerus fracture diagnosis from January 1, 2011-December 31, 2011 (ICD-9 Diagnosis codes: 812.00, 812.01, 812.02, 812.09, 812.10, 812.11, 812.12, 812.13, 812.19) (Index diagnosis) | 130,959 |
| No Part B carrier, outpatient, or MEDPAR claims with proximal humerus fracture diagnosis in 365-days before the index diagnosis in 2011 | 107,838 |
| Shoulder x-ray claim (HCPCS codes: 73000, 73010, 73020, 73030, 73050, 73060) in Part B carrier or outpatient revenue center claims within 7 days of index diagnosis (x-ray claim can occur before or after index diagnosis) | 95,229 |
| No Part B carrier, outpatient, or MEDPAR claims with a diagnosis of clavicle or hip fracture within 7 days of index diagnosis | 86,147 |
| No Part B carrier, outpatient, or MEDPAR claims with total joint replacement procedure in 365-days before the index diagnosis in 2011 | 85,841 |
| Age 66+ at index diagnosis | 84,589 |
| Located within continental United States or Hawaii | 84,399 |
| Continuously enrolled in Medicare Parts A and B and never enrolled in HMO, from 365-days prior to index to 365-days after index diagnosis | 77,075 |
| Complete HRR data | 77,053 |

| *Medicare ARCT Cohort Size by Study Sample Inclusion Criteria* | |  |
| --- | --- | --- |
| **Inclusion Criteria** | **N** | |
| Medicare Part B “Carrier” claim with shoulder-related diagnosis in 2011 (Index diagnosis) | 2,525,519 | |
| No carrier claim with shoulder-related diagnosis in 365-days before index diagnosis in 2011 | 1,871,294 | |
| Continuously enrolled in Medicare Parts A and B, never enrolled in HMO, from 365-days prior to index to 104-days after index | 1,598,175 | |
| Aged 66+ at index | 1,588,184 | |
| No claim indicating ED or ambulance use within 1 day of index date | 1,290,052 | |
| Claim indicating MRI within 90-days after index | 144,487 | |
| RCT diagnosis within 14-days after first MRI | 65,220 | |
| No cervical spine pain, scapular pain, glenohumeral arthritis, humerus fracture, inflammatory arthritis, adhesive capsulitis, or dementia during period 365-days prior to index to 104 days after index date | 33,336 | |
| Complete data available for geography-based measures | 32,163 | |

**Appendix A2: Full Description of Variables**

| **Variable/Concept** | **Time Frame** | **Source** | **Qualifying Codes** | **Data Files** |
| --- | --- | --- | --- | --- |
| Emergency department and ambulance use near index shoulder complaint | Index date and day prior | Revenue Center codes and Berenson-Eggers Type of Service Codes | (Revenue Center Codes: 0450, 0451, 0452, 0456, 0459, 0981, 0540, 0541, 0542, 0543, 0544, 0545, 0546, 0547, 0548, or 0549) and (Berenson-Eggers Type of Service Codes beginning with: M3 or A01) | Outpatient revenue center, Carrier |
| MRI of the upper extremity or orbit, face and neck | Within 90 days of index date | HCPCS codes | **70540, 70542, 70543, 73218, 73219, 73220, 73221, 73222, or 73223** | Carrier (Part B Physician Claims), Outpatient revenue center |
| Diagnosis of ARCT | Within 14-days of the earliest dated MRI | ICD-9 Diagnosis Code | **727.61, 840.3, or 840.4** | Carrier (Part B Physician Claims), **Outpatient base claims** |
| Diagnosis of PHF | Within 7-days of the earliest dated X-ray | ICD-9 Diagnosis Code | **812.00, 812.01, 812.02, 812.09, 812.10, 812.11, 812.12, 812.13, 812.19 (any DX on the claim)** | Carrier (Part B Physician Claims), **Outpatient base claims** |
| Surgical ARCT Repair | Surgery claim within the 104-day treatment exposure period after the MRI and diagnosis of ARCT and no earlier or concurrent claim for PT | ICD-9 Procedure codes or HCPCS codes | **with ICD-9 procedure codes (80.81, 81.88, 81.82, 81.83 and 83.63) or HCPCS codes (23410, 23412, 23420, 23472, 29827, 29820, 29822, 29823, 29824, 29826, 29828)** | Carrier, Outpatient revenue center, Outpatient base claims, MEDPAR |
| Surgical PHF Repair | Surgery claim within the 60-day treatment exposure period after the MRI and diagnosis of an ARCT and no earlier or concurrent claim for PT | ICD-9 Procedure codes or HCPCS codes | **HCPCS codes (23630, 23615, 23670, 23680, 23472, 23470, 23616)** |  |
| Patient Age | On index date | Date of birth filed with Medicare |  | Medicare Beneficiary A/B Summary File |
| Frailty Index (FRI) | All claims over period one year prior to index date | Chrischilles EA, Schneider KM, Wilwert J, Lessman G, O’Donnell B, et al. “Beyond comorbidity: Expanding the definition and measurement of complexity among older adults using administrative claims data.” *Medical Care. 2014;* 52(3), S75-84.  Chrischilles EA, Schneider KM, Shroeder M, Letuchy E, Wallace RB, Robinson JG. “Association of pre-admission functional status with use and effectiveness of secondary prevention medications in elderly survivors of acute myocardial infarction.” *J of the American Geriatrics Society*, 2016; 64(3): 526-535. DOI: 10.1111/jgs.13953 | | |
| Charlson Comorbidity Index (CCI) | All claims over period one year prior to index date | Charlson ME, Pompei P, Ales KL, et al. A new method of classifying prognostic comorbidity in longitudinal studies: development and validation. Journal of chronic diseases 1987;40:373-383 | | |
| Sum Total Payments made by Medicare and Beneficiary to Providers in 365 days prior to index | All claims over period one year prior to index date | Line Payment Amounts | N/A | Inpatient, Outpatient, Durable Medical Equipment, Home Health, Carrier (Physician services), skilled nursing |
| Gender | 2011 | Gender filed with Medicare in 2011 | N/A | Medicare Beneficiary A/B Summary File |
| Race | 2011 | Race filed with Medicare in 2011 | N/A | Medicare Beneficiary A/B Summary File |
| Medicaid Dual Eligible Status | Index Month | Dual-eligibility status in month of index (not dual eligible, partial dual-eligible, or fully dual-eligible) | Dual-Status code of 01 through 06, or 08. | Medicare Beneficiary Part D Components File |
